# Misdiagnosis of Paget’s Disease of Bone in a Congenital Generalized Lipodystrophy Patient: Case Report

**DOI:** 10.3389/fendo.2021.683697

**Published:** 2021-06-28

**Authors:** Erika Bastos Lima Freire, Mayara Ponte Madeira, Grayce Ellen da Cruz Paiva Lima, Virginia Oliveira Fernandes, Lindenberg Barbosa Aguiar, João Paulo Uchoa Fontenele, Ana Paula Dias Rangel Montenegro, Thyciara Fontenele Marques, Renan Galvão Ozório, Catarina Brasil d’Alva, Renan Magalhães Montenegro

**Affiliations:** ^1^ Clinical Research Unit, Walter Cantídio University Hospital, Federal University of Ceará, Fortaleza, Brazil; ^2^ Department of Clinical Medicine, Federal University of Ceará, Fortaleza, Brazil; ^3^ Health Sciences Center, Christus University Center (UNICHRISTUS), Fortaleza, Brazil; ^4^ Health Sciences Center, University of Fortaleza, (UNIFOR) Fortaleza, Brazil; ^5^ Department of Community Health, Federal University of Ceará, Fortaleza, Brazil; ^6^ Department of Radiology, Federal University of Ceará, Fortaleza, Brazil; ^7^ Pathology Department, Dr. Cesar Cals de Oliveira General Hospital, Fortaleza, Brazil; ^8^ Health Sciences Center, Medical Faculty of Juazeiro do Norte, Juazeiro do Norte, Brazil

**Keywords:** Berardinelli-Seip syndrome, Paget’s Disease of bone, congenital generalized Lipodystrophy patient, Bone, Lipodystrophy, case report

## Abstract

Paget’s disease of bone (PDB) is a common skeleton disorder in which the diagnosis is suggested by radiological analyses. Congenital generalized lipodystrophy (CGL) is a rare, but a radiologic differential diagnosis of Paget’s disease. Patients present total or almost total lack of subcutaneous adipose tissue, leptin deficiency, and precocious ectopic lipid accumulation, which lead to intense insulin resistance, poorly controlled diabetes mellitus, and hypertriglyceridemia. CGL subtypes 1 and 2 present sclerosis and osteolytic lesions that can resemble “pagetic” lesions. The clinical correlation is, therefore, essential. We report a CGL patient with bone lesions in which the radiographic findings led to a misdiagnosis of PDB. This case report brings awareness to CGL, a life-threating condition. Its early recognition is essential to avoid clinical complications and premature death. Therefore, it is important to consider CGL as PDB’s differential diagnosis, especially in countries with high prevalence of this rare disease, such as Brazil.

## Introduction

Paget’s disease of bone (PDB) was first described in 1876 and was named “osteitis deformans” because of its most impressive feature at that time (1). Next to osteoporosis, it is the most frequent metabolic bone disease (2). It is characterized by focal increase in bone turnover followed by a high rate of bone formation. This results in the bone’s microarchitecture alterations, which can lead to skeletal deformity and fractures (3, 4). Along with the intense osteoblastic and osteoclastic performance, affected bones present marrow fibrosis and increased vascularity (5). Its epidemiology varies among countries. A meta-analysis in 2013 showed the highest prevalence rate of 5.4% in United Kingdom and a drop in global incidence over the recent years (2). Epidemiological data in South America are scarce. In Brazil, the most important prevalence study was conducted in a city located in the northeast region, which had European colonization, and the rates were comparable to those in southern Europe (6, 7). The etiology is not fully known; however, it seems that genetic and environmental factors play a role (4, 5). It is slightly more frequent in males, and its major risk factor is probably aging (8). It is very rare before the age of 50 years (4, 9). Most PDB patients are asymptomatic and diagnosed after an abnormal bone imaging exam or an incidental elevated serum alkaline phosphatase (AP) performed to evaluate another clinical disorder, although it can be normal in mild cases (4, 8). In low disease activity, it is recommended to assess specific markers of bone turnover, such as Procollagen I Intact N-Terminal (P1NP) and specific AP (4). The clinical manifestations depend on which bones are affected and can include enlargement of the skull, augmentation, and bowing of the thighs (femurs) and lower legs (tibias) (4). Bone pain is the most common complaint in symptomatic patients (10). It can be caused by osteolytic lesions, secondary osteoarthritis, fractures, nerve compression syndromes, or even bone malignancy (4, 8). Deformity is a frequent presentation in more severe cases, mainly in long bones (femur and tibia) and the skull (11, 12). Fractures can also occur in affected bones.

The radiological findings correlate to the phase of the disease. Initially, osteoclastic activity is increased, leading to osteolytic lesions in skull and long bones, in which radiolucency areas can assume the pattern of “blade of grass” (13). In the skull, lytic lesions can be present in the occipital and frontal bones (14). The high rate of bone formation comes after the initial phase, resulting in sclerosis along with the lytic lesions. In the final and inactive phase, sclerosis is the predominant feature (13). Although any bone can present both sclerosis and lytic lesions, the axial skeleton is more frequently affected [pelvis (70%), lumbar spine (53%), skull (42%)]. In the appendicular sites, femur (55%) and tibia (32%) are most commonly involved (4, 15–17).

PDB diagnosis is usually suggested by radiological analyses or scintigraphy, and the most notable feature is the enlargement of the bone (12). Hence, knowledge of PDB’s radiologic differential diagnosis is very important. The most common ones are fibrous dysplasia, chronic osteomyelitis, and metastatic lesions (12, 18). A rare PDB differential diagnosis is congenital generalized lipodystrophy (CGL), also known as Berardinelli-Seip syndrome (19).

CGL is a rare autosomal recessive disease, more frequently found in parental consanguinity cases. It was first described by a Brazilian physician in 1954, and only about 500 cases have been reported since then (100 of those described in Brazil) (20, 21). Its incidence is higher in Lebanon, Portugal, Oman, and Brazil, in which the northeast region hosts the majority of cases (22, 23). CGL is caused by mutations of the genes responsible for the adipocyte development, which lead to total or almost total lack of subcutaneous adipose tissue (since birth or early childhood), leptin deficiency, and precocious ectopic lipid accumulation. Patients present intense insulin resistance, poorly controlled diabetes mellitus (around 45% diagnosed in pubertal years), and hypertriglyceridemia (21). Besides metabolic disorders, CGL patients develop liver disease, early cardiac abnormalities, and infectious complications, which result in greatly reduced life expectancy (24, 25).

CGL is categorized in four subtypes according to the affected gene (24). CGL subtype 1 (CGL1) and 2 (CGL2), associated with mutations in *AGPAT2* and *BSCL2*, correspond to almost 95% of cases (21). Clinically, CGL patients present hyperphagia, prognathism, acanthosis nigricans, generalized lack of subcutaneous fat, muscle hypertrophy, phlebomegaly, and umbilical protrusion. Some clinical features point to a specific subtype of the disease. CGL1 patients often present acromegaloid facies, preserved fat in the palms and sole, as opposed to CGL2, and more frequently evolve with focal lytic lesions in long bones, which can resemble PDB’s skeletal findings (19, 21, 26).

We report a CGL1 patient with bone lesions in which the radiographic findings led to a misdiagnosis of PDB. Informed consent was obtained from the subject of this study.

## Case Report

A 41-year-old male, born out of a third-degree consanguineous marriage in the state of Ceará (northeast region of Brazil), was referred to our University Hospital, a reference center in lipodystrophy (Brazilian Group for the Study of Inherited and Acquired Lipodystrophies-BRAZLIPO, Federal University of Ceará) because of suspected lipodystrophy and previous PDB diagnosis. He reported five cousins diagnosed with CGL and no familiar history of bone disease.

At age of 15 years, the patient noticed generalized lack of subcutaneous fat, unlike his parents and two siblings. Two years later, he was diagnosed with diabetes mellitus (DM). He had both mild hypertriglyceridemia and hyperglycemia. At age of 20 years, he was started on oral antidiabetics and low-carb diet. He lost follow-up and resumed medical care at the age of 28 years, when insulin therapy was initiated, leading to good glycemic control. At 33 years old, he presented progressive right hip pain that irradiated to the lower limbs, which was improved with non-steroidal anti-inflammatory and became bilateral 5 years later, which is when he sought medical help. Hip computed tomography (CT) was performed to investigate the worsening of the symptom, which showed multiple foci of sclerotic lesions, a 2-cm bone cyst on right femoral head, and another 3-cm cyst on the left femoral head. At that time, PDB was suspected, and the skull, lower limbs, upper limbs, and hip x-rays undertaken were normal. Serum C-linked C-telopeptide of type 1 collagen (CTX) was 0.245 ng/ml (range, 0,016–0,584 ng/ml), and AP concentration was 59 ng/ml (range, 40–129 ng/ml). Bone-specific AP, P1NP, and bone scintigraphy were not undertaken, once the previous follow-up was conducted in a poor region of the state of Ceará.

Atypical PDB was then suspected, and an iliac crest biopsy was performed. Histopathological analysis, at the time, was thought to indicate increased osteoclastic activity; therefore, the diagnosis of PDB was suggested.

Three years after the initial investigation, because of the worsening of the hip pain and the suspected lipodystrophy, the patient was referred to our center in lipodystrophy. He brought only medical records but no previous radiological images. Our physical examination revealed a BMI of 22.5 kg/m^2^, generalized lack of subcutaneous fat with preserved fat in the palms and soles, muscle hypertrophy, phlebomegaly, and umbilical hernia. He did not have acanthosis nigricans or signs of bone deformity.

**Figure 1:**
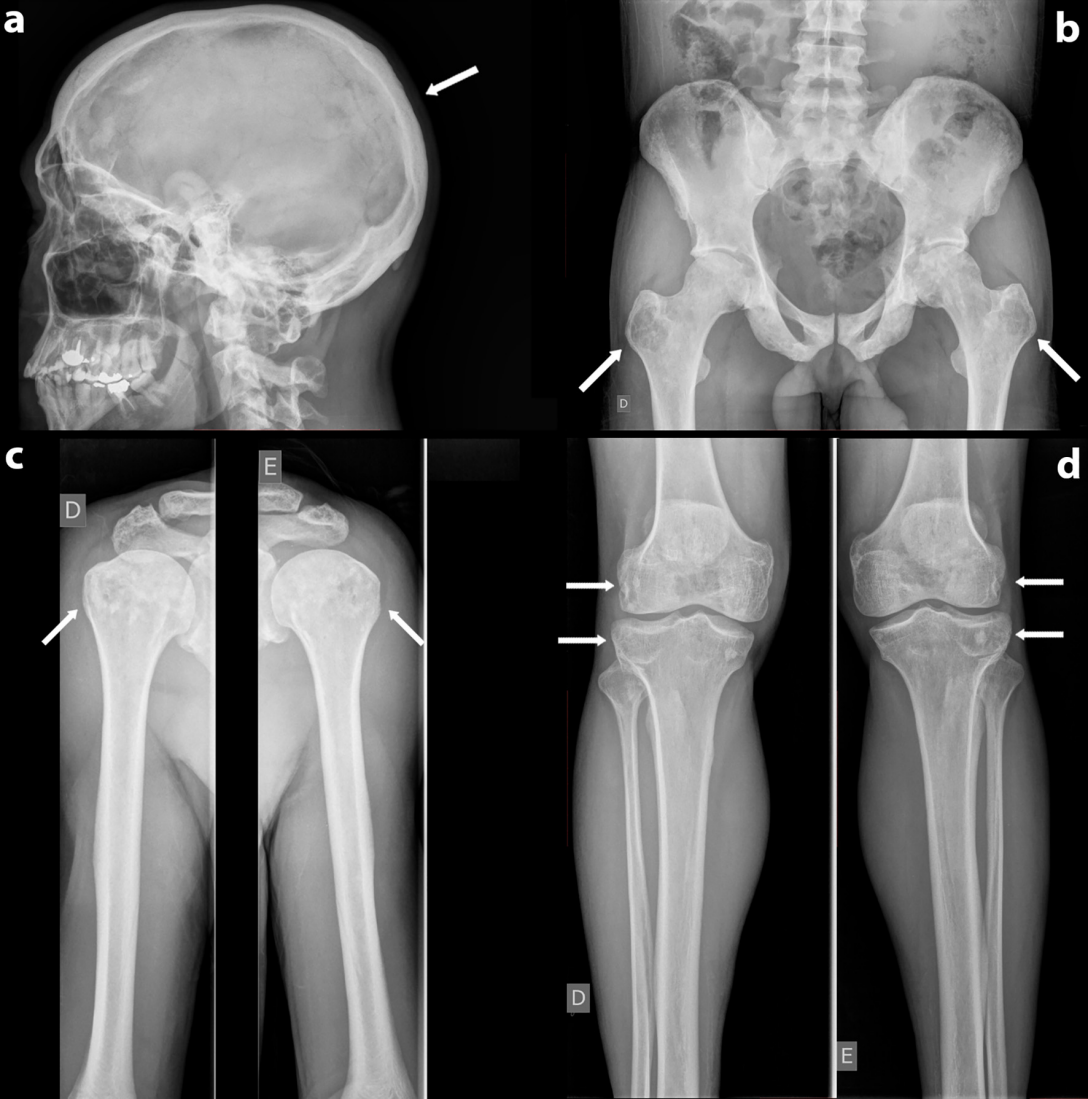
**(A)** Normal skull radiography. **(B)** Anteroposterior radiography of the pelvis Symmetric radiolucency with cystic aspect in greater trochanter bilaterally. **(C)** Anteroposterior radiography of upper limbs- heterogeneous bone density in humeral heads bilaterally with the presence of sclerosis and radiolucent areas. **(D)** Anteroposterior radiography of lower limbs-pseudo-poikilosis (patchy sclerotic lesions) and radiolucency in distal metaphysis of right and left femur and in proximal region of tibia bilaterally.

New biochemical parameters showed leptin of 0.3 ng/ml (range, 2.0–5.6 ng/mL), hyperlipidemia (serum cholesterol, 188 mg/dl; triglycerides, 524 mg/dl; high-density lipoproteins [HDL], 26 mg/dl), glycated hemoglobin (HbA1C) of 7.3%, fasting blood glucose of 108 mg/dl, and serum AP of 218 U/L (range, 65–300 U/L). A new skeletal survey was performed. Skull x-ray revealed a homogeneous increase in cranial cap density. There was no thickening of the cranial vault (**Figure 1A**). There was no evidence of focal sclerosis or trauma. Cervical, thoracic, and lumbar spine x-rays also revealed increased vertebrae density without signs of focal trauma. Upper-limb x-rays showed heterogeneous density in humeral heads bilaterally, with the presence of sclerosis and radiolucent areas (**Figure 1C**). This pattern was also present in pelvis, distal metaphysis of right and left femur and in proximal region of tibia (**Figures 1B, D**). In the hip x-ray, there was a sclerosis in the pubis, ischius, and iliaco, as well as pseudo-osteopoikilosis (patchy sclerotic lesions) in the femur head bilaterally. Symmetric radiolucency with cystic aspect was demonstrated in greater trochanter bilaterally (**Figure 1B**). The hands radiography was normal. There were no signs of deformity or fractures in the skeletal survey. Bone mineral density (BMD) was assessed by DXA (dual energy x-ray absorptiometry, GE Prodigy Lunar). It showed an L1-L4 Z-score of +3.6 (BMD 1,649 g/cm^2^) and left femoral neck Z-score of +2.4 (BMD 1,343 g/cm^2^).

CGL subtype 1 was confirmed by molecular analysis. The patient presented a homozygous deletion of nucleotides 317 to 588, resulting in the absence of exons 3 and 4 (c.del.317-588, p.del.Gly106-Gln196) in the *AGPAT2* gene.

**Figure 2:**
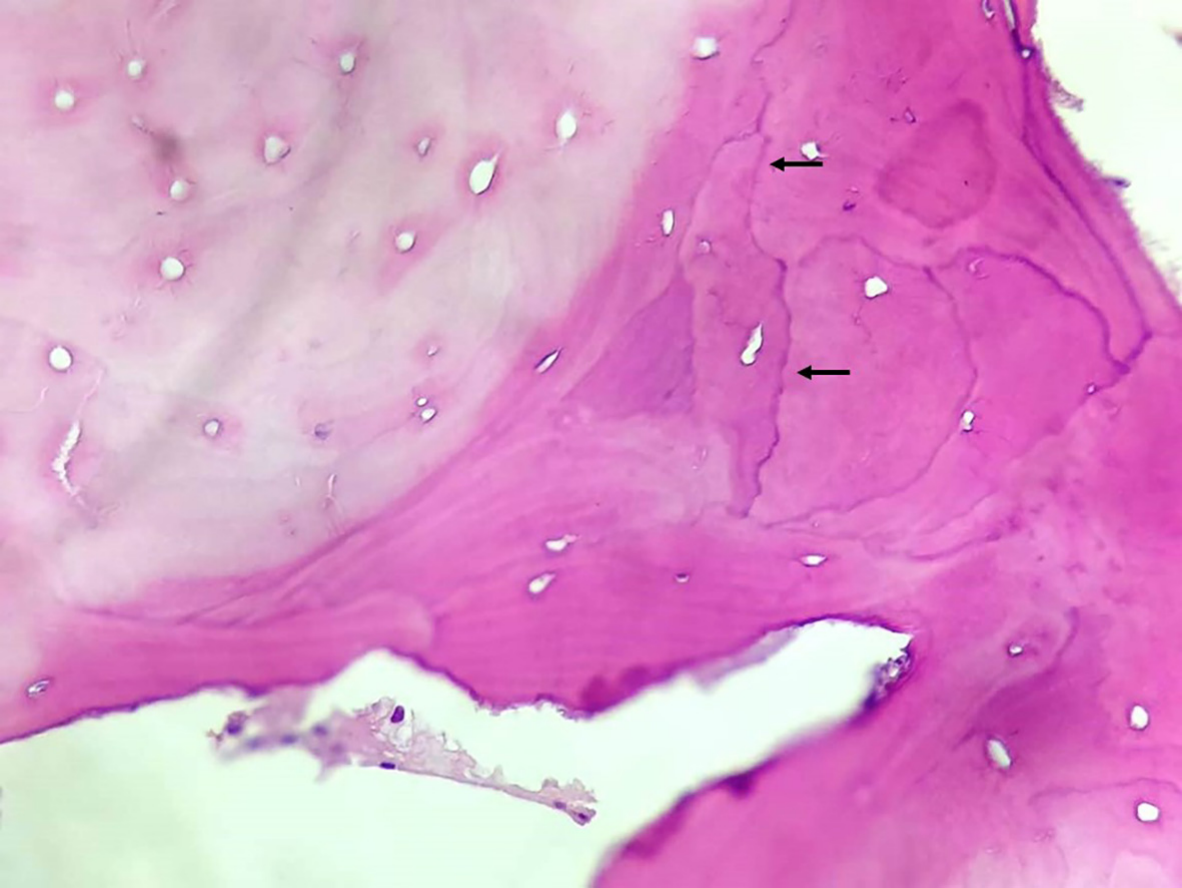
Bone tissue with signs of remodeling. HE stain, magnification 200×… See cement lines (arrows).

Once PDB misdiagnosis was suspected, we requested a pathology review. Because CGL is a rare disorder, the pathologist was advised about CGL diagnosis and its radiologic features before the new evaluation. The histopathological analysis revealed a non-lamellar bone tissue (immature type) with bundles of collagens arranged in multiple directions. There were no of signs of osteoclastic activity nor neoplasm (**Figure 2**).

## Discussion

We describe a patient with clinical features of CGL born out of a consanguineous marriage in the Northeast region of Brazil, where there is a high prevalence of CGL, probably because of the higher frequency of parental consanguinity. A large series of CGL identified consanguineous parental marriages as its most important risk factor (27). Our patient had not only CGL clinical phenotype but also insulin-dependent DM, hypertriglyceridemia, and low leptin levels, which are common CGL characteristics. The late CGL diagnosis is probably because of the rarity of this disease and because of the fact that, in males, classic lipodystrophic features, such as muscular hypertrophy, phlebomegaly, and acromegaloid facies, are less suspicious than in women. Therefore, health professionals should be aware about this condition to make an early diagnosis (24). The patient also had preserved mechanical adipose tissue in the hands and soles, suggestive of CGL 1, in which bone radiological findings (radiologically similar to “pagetic” lytic lesions) are more commonly found (21). The CGL 1 diagnosis was confirmed by the identification of the *AGPAT2* gene mutation.

An extensive review of longitudinal studies and standard case reports showed that CGL1 and CGL2 have increased bone mass, bone cysts, and sclerosis, which are findings present in our patient (28). Focal lytic lesions in long bones, more frequently found in CGL1, and also present in CGL2, typically evolve after puberty and are a risk factor for fractures. Patients must be evaluated and advised to avoid contact sports if cystic lesions are present (21, 29). The largest cohort so far published found that bone radiological findings in CGL patients include sclerosis, osteolytic lesions, and pseudo-osteopoikilosis (19). Bone sclerosis is diffuse in axial and appendicular skeleton, as well as trabecular and cortical bone. Osteolytic lesions are bilateral and symmetric, mostly in appendicular skeleton and pelvis. Hands are frequently involved (19). Pseudo-osteopoikilosis is a little less frequent and mostly found in the epiphysis and metaphysis of the iliac bones and proximal femurs (19). Histological evaluation of CGL cystic lesions specimens shows occasional increased osteoclastic activity and enhanced cement lines, demonstrating bone remodeling and resorption notches with the delimitation of true cysts containing tissue rich in blood (30, 31).

The misdiagnosis was probably influenced by the fact that PDB is a common skeletal disease, with a much higher prevalence than CGL. He was 41 years old, which is a relatively young age for the diagnosis of PDB as it is very rarely diagnosed before age 50 (4). Patient started to experience the symptoms (hip pain) at the age of 33 years. In many cases, it can be difficult to distinguish “pagetic” bone pain from its differential diagnoses, such as musculoskeletal disorders, because it is not a specific PDB presentation. Once it is a common symptom, it conducted to a PDB suspicion (32). The patient had increased bone density without signs of focal trauma in axial skeleton. In appendicular sites, there were signs of sclerosis, pseudo-osteopoikilosis, as well as radiolucent areas with symmetrical bone cysts in greater trochanter bilaterally, which are common findings in CGL1. Bone involvement in PDB is usually asymmetric, and true cysts have not been described in this disease (10, 12–14). He had no lytic lesions on the skull, enlargement of bone on radiological analyses, deformity or fractures, which along with repeatedly normal AP levels, led us to suspect the misdiagnosis of PDB.

Unlike PDB, bone lesions rarely lead to CGL diagnosis. Nevertheless, Hasani-Ranjbar et al. (33) described a 25-year-old female referred to an endocrinology center to investigate acral enlargement, hepatomegaly, and bone lesions (sclerotic and cystic). The radiologic findings helped to suspect and establish a late CGL1 diagnosis.

Once an atypical PDB case was suspected, iliac crest biopsy was performed. According to the Endocrine Society clinical practice guideline, experienced radiologists and physicians generally have no difficulty in recognizing PDB’s lesions, and a need for a biopsy is rare, although it can be helpful in atypical cases (4). The initial histopathological analysis was thought to indicate signs of increased osteoclastic activity, therefore, the diagnosis of PDB was suggested. The histological analysis of 754 PDB patients showed enlarged spongious bone with a significant increase in osteoblastic and osteoclastic activity (34). Our pathologist’s histopathological review had a different description than the first assessment. It revealed only bone tissue with signs of bone remodeling. The signs of increased osteoclastic activity previously described were not observed, although the acellularity of the bone could be an artifact of the way the slide was prepared. Bone remodeling is a very nonspecific finding that can be found in any condition with high bone metabolism, like fractures, osteomyelitis, neoplasms, PDB, and CGL. The clinical correlation is, therefore, essential. Because the clinical and molecular diagnoses were stablished before the new histopathological analysis, our pathologist was advised about CGL and its radiologic features. We believe that the pathologist that performed the first analysis was influenced by the clinical diagnostic hypothesis of PDB, which is a much more frequent disease.

This case report brings awareness to not only a rare, but also important, PDB differential diagnosis, which is CGL. Bone radiologic findings can be similar, and the histological evaluation of both disorders can resemble, which makes the knowledge of CGL and its clinical presentation very important to the physicians that study PDB and its differential diagnosis.

CGL is a life-threating condition, and its early recognition is essential to avoid clinical complications and premature death. Therefore, in suspected cases, an evaluation in a reference center in lipodystrophy should be considered.

In conclusion, misdiagnosis of PDB can occur in CGL, mainly in subtype 1 patients, because of the cystic lesions. Therefore, the differential diagnosis of this rare disease is important to consider, especially in countries with high prevalence, such as Brazil.

## Data Availability Statement

All datasets presented in this study are included in the article/supplementary material.

## Ethics Statement

The studies involving human participants were reviewed and approved by The Ethics Committee of University Hospital of Ceará Federal University. The patients/participants provided their written informed consent to participate in this study.

## Author Contributions

All authors contributed to data analysis, drafting, and revising the article, gave final approval of the version to be published, and agree to be accountable for all aspects of the work.

## Conflict of Interest

The authors declare that the research was conducted in the absence of any commercial or financial relationships that could be construed as a potential conflict of interest.
